# Supplementary material for: Determinants of promoter and enhancer transcription directionality in metazoans
Source: Nat Commun. 2018 Oct 26;9:4472. doi: 10.1038/s41467-018-06962-z (PMC6203779; doi:10.1038/s41467-018-06962-z)
Supplement: Supplementary file 1 — Supplementary Info [file 41467_2018_6962_MOESM1_ESM.pdf]

Supplementary Information

Ibrahim et al.

Determinants of Promoter and Enhancer Transcription Directionality in Metazoans

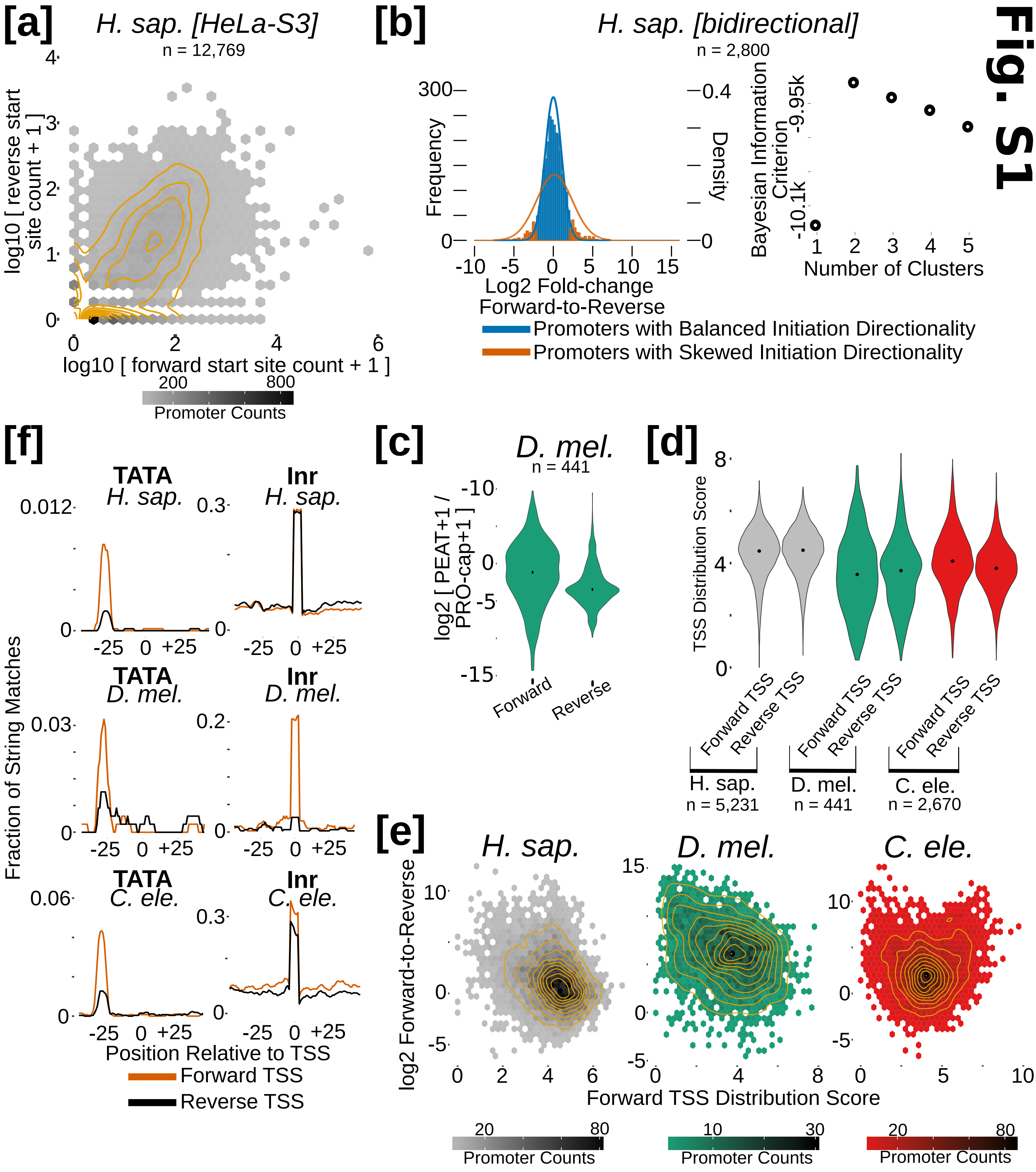
[a]
H. sap. [HeLa-S3]
n = 12,769
log10 [reverse start site count + 1]
log10 [forward start site count + 1]
200
800
Promoter Counts
[b]
H. sap. [bidirectional]
n = 2,800
Frequency
Density
Log2 Fold-change Forward-to-Reverse
Bayesian Information Criterion
-9.95k
-10.1k
Number of Clusters
Promoters with Balanced Initiation Directionality
Promoters with Skewed Initiation Directionality
[c]
D. mel.
n = 441
log2 [PEAT+1 / PRO-cap+1]
Forward
Reverse
[d]
TSS Distribution Score
Forward TSS
Reverse TSS
H. sap.
n = 5,231
D. mel.
n = 441
C. ele.
n = 2,670
[e]
H. sap.
D. mel.
C. ele.
log2 Forward-to-Reverse
Forward TSS Distribution Score
20
80
Promoter Counts
10
30
Promoter Counts
20
80
Promoter Counts
[f]
TATA
H. sap.
Inr
H. sap.
TATA
D. mel.
Inr
D. mel.
TATA
C. ele.
Inr
C. ele.
Fraction of String Matches
Position Relative to TSS
Forward TSS
Reverse TSS

Fig. S1

Supplementary Figure 1. Characteristics of divergent promoters.

a. A contour/hexbin scatter plot of forward versus reverse direction 5'-GRO-Seq counts plotted for promoter NDRs defined by DNaseI-seq in *H. sapiens* HeLa cells. **b.** Mixture models (left) and Bayesian Information Criterion analysis of cluster numbers (right) for forward/reverse GRO-cap count ratios for bidirectional (stable-stable) promoter NDRs in *H. sapiens* containing significant initiation in at least one direction (n = 2800). A pseudo count of 1 was added to numerators and denominators. Lines represent density of the theoretical Gaussian distributions learned from the data, histograms represent observed ratios. Forward and reverse directions were assigned randomly. **c.** Forward and reverse PEAT-to-PRO-cap count ratio distributions for divergent *D. melanogaster* promoter NDRs containing significant forward and reverse TSSs. A pseudo-count of 1 was added to both numerators and denomenators. Black dots represent median values. **d.** Forward and reverse TSS distribution entropy scores for promoter NDRs containing significant forward and reverse TSSs. Black dots represent median values. **e.** Contour/hexbin scatter plots of forward TSS distribution scores versus forward-to-reverse initiation ratio. **f.** Initiator and TATA-box consensus sequence string matches relative to forward and reverse TSS modes for promoter NDRs containing significant forward and reverse TSSs.

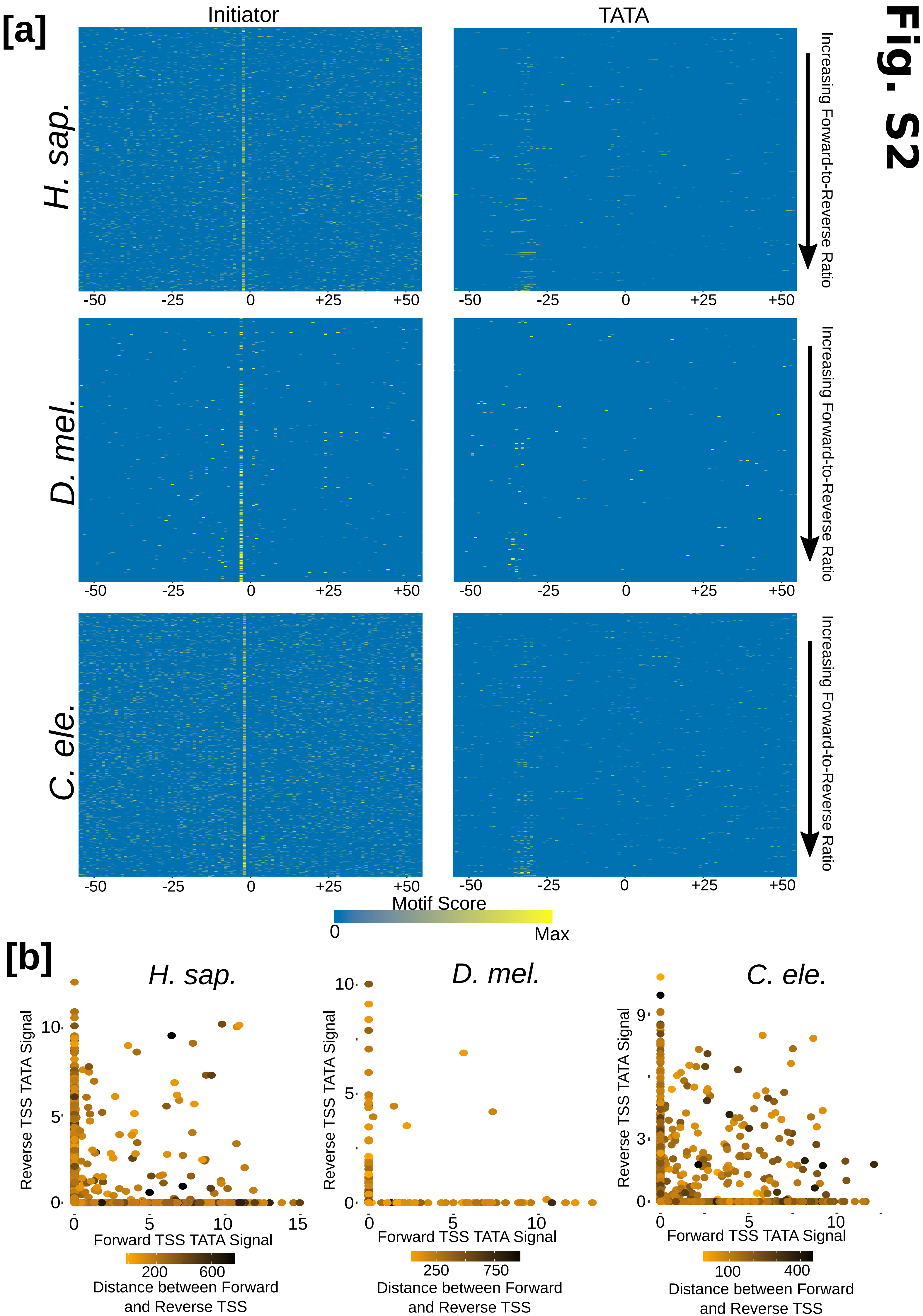
Fig. S2
[a]
Initiator
TATA
H. sap.
D. mel.
C. ele.
-50
-25
0
+25
+50
Increasing Forward-to-Reverse Ratio
Motif Score
0
Max
[b]
H. sap.
D. mel.
C. ele.
Reverse TSS TATA Signal
Forward TSS TATA Signal
Distance between Forward and Reverse TSS
200
600
250
750
100
400

Supplementary Figure 2. Distribution of core promoter motifs in divergent promoters.

a. Motif scans of *Initiator and TATA* core promoter motifs for promoter NDRs with significant initiation in both the forward and reverse directions. Heatmaps are sorted by increasing forward-to-reverse initiation ratio and scaled to make the maximum color equal to the 0.999 quantile of all scores. **b.** Scatter plots for maximum TATA motif signal in a -45 to -25 window upstream of TSSs. Forward versus reverse signals are displayed. Color of the dots represents the distance between the forward and reverse TSSs. Distance colors for *H. sapiens* and *C. elegans* were scaled so that the maximum is 0.99 quantile of all distances (767 basepairs and 453 basepairs respectively).

Fig. S3

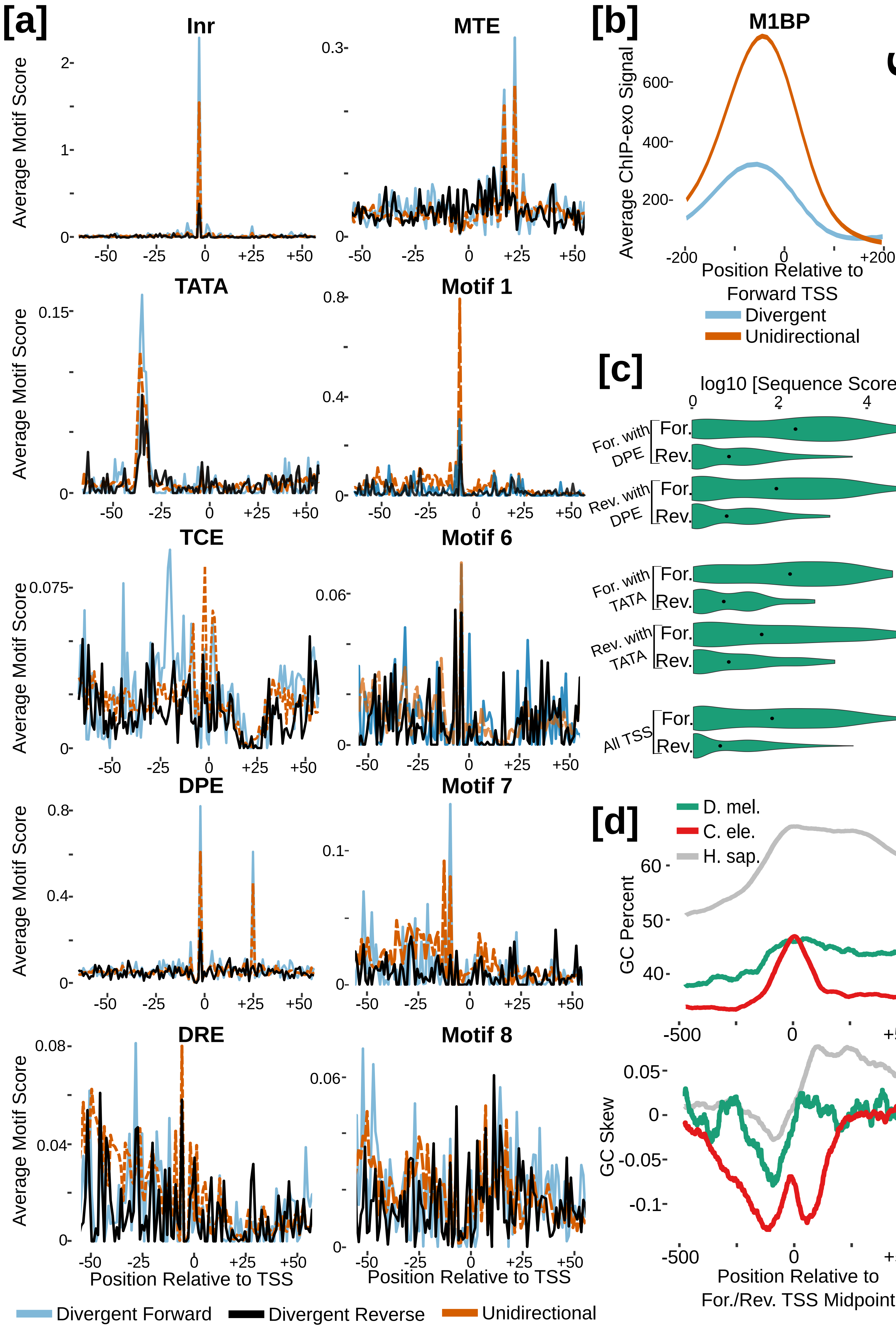
[a]
Inr
MTE
TATA
Motif 1
TCE
Motif 6
DPE
Motif 7
DRE
Motif 8
Average Motif Score
Position Relative to TSS
Divergent Forward
Divergent Reverse
Unidirectional
[b]
M1BP
Average ChIP-exo Signal
Position Relative to Forward TSS
Divergent
Unidirectional
[c]
log10 [Sequence Score]
For. with DPE
Rev. with DPE
For. with TATA
Rev. with TATA
All TSS
For.
Rev.
[d]
D. mel.
C. ele.
H. sap.
GC Percent
GC Skew
Position Relative to For./Rev. TSS Midpoint

Supplementary Figure 3. Relationship of *D. melanogaster* core promoter motifs to transcription initiation directionality.

a. Motif scans of *D. melanogaster* core promoter motifs for promoter NDRs with significant initiation in the forward direction only (Unidirectional, orange; n = 2764) and promoter NDRs with significant forward and reverse initiation levels (Divergent Forward, blue; Reverse, black; n = 441). **b.** Average ChIP-exo signal for Motif 1 Binding Protein (M1BP) at unidirectional (orange, n = 2764) and divergent (blue, n = 441) promoter NDRs. **c.** Sequence model scores for *D. melanogaster* divergent promoter regions whose forward- or reverse-directed core promoter sequences are positive for TATA or DPE motifs (see Methods; For. with DPE, n=150; Rev. with DPE, n=115; For. with TATA, n=55; Rev. with TATA, n=50; all Divergent promoters, n=441). **d.** Positional averaged analyses of GC percentage (top) and GC skew (Bottom) in 50 bp sliding windows relative to midpoints between forward and reverse TSS modes for promoter NDRs containing significant forward and reverse TSSs.

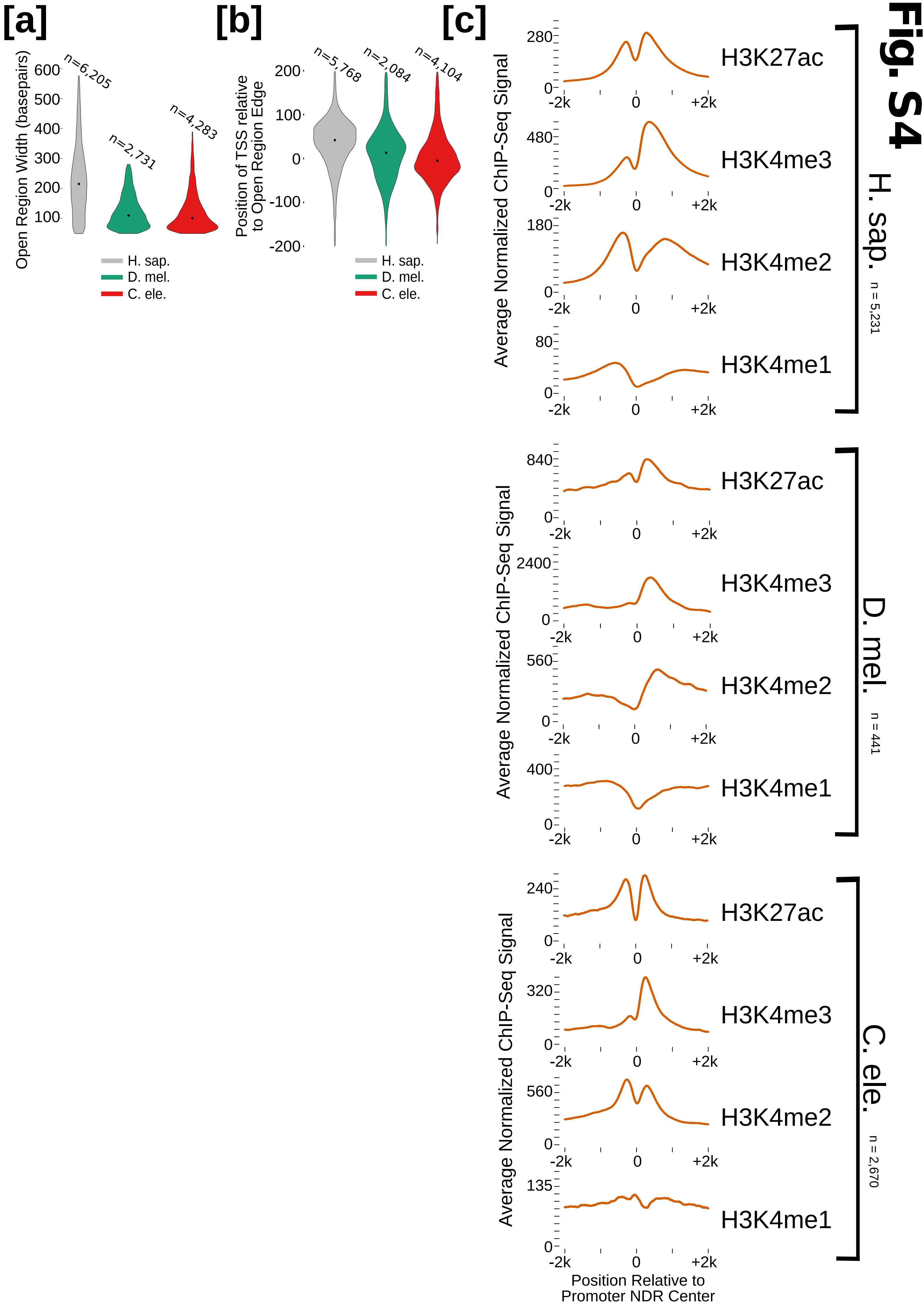

Fig. S4
[a]
[b]
[c]
Open Region Width (basepairs)
600
500
400
300
200
100
n=6,205
n=2,731
n=4,283
H. sap.
D. mel.
C. ele.
Position of TSS relative to Open Region Edge
200
100
0
-100
-200
n=5,768
n=2,084
n=4,104
H. sap.
D. mel.
C. ele.
H. sap.
n = 5,231
Average Normalized ChIP-Seq Signal
H3K27ac
280
H3K4me3
480
H3K4me2
180
H3K4me1
80
0
-2k
0
+2k
D. mel.
n = 441
Average Normalized ChIP-Seq Signal
H3K27ac
840
H3K4me3
2400
H3K4me2
560
H3K4me1
400
C. ele.
n = 2,670
Average Normalized ChIP-Seq Signal
H3K27ac
240
H3K4me3
320
H3K4me2
560
H3K4me1
135
Position Relative to Promoter NDR Center

Supplementary Figure 4. Variation of promoter chromatin architecture across species.

a. Expressed promoter NDR width distribution based on ATAC-Seq peak calls. **b.** Distributions of TSS positions relative to NDR edge (negative numbers indicate the TSS is downstream of the NDR edge and outside the NDR ATAC-seq peak, positive numbers indicate the TSS is upstream of the NDR edge and inside the NDR ATAC-Seq peak). Black dots represent median values. **c.** Average analyses of histone PTM ChIP-Seq signal relative to midpoints of promoter NDRs containing significant forward and reverse TSSs.

Fig. S5

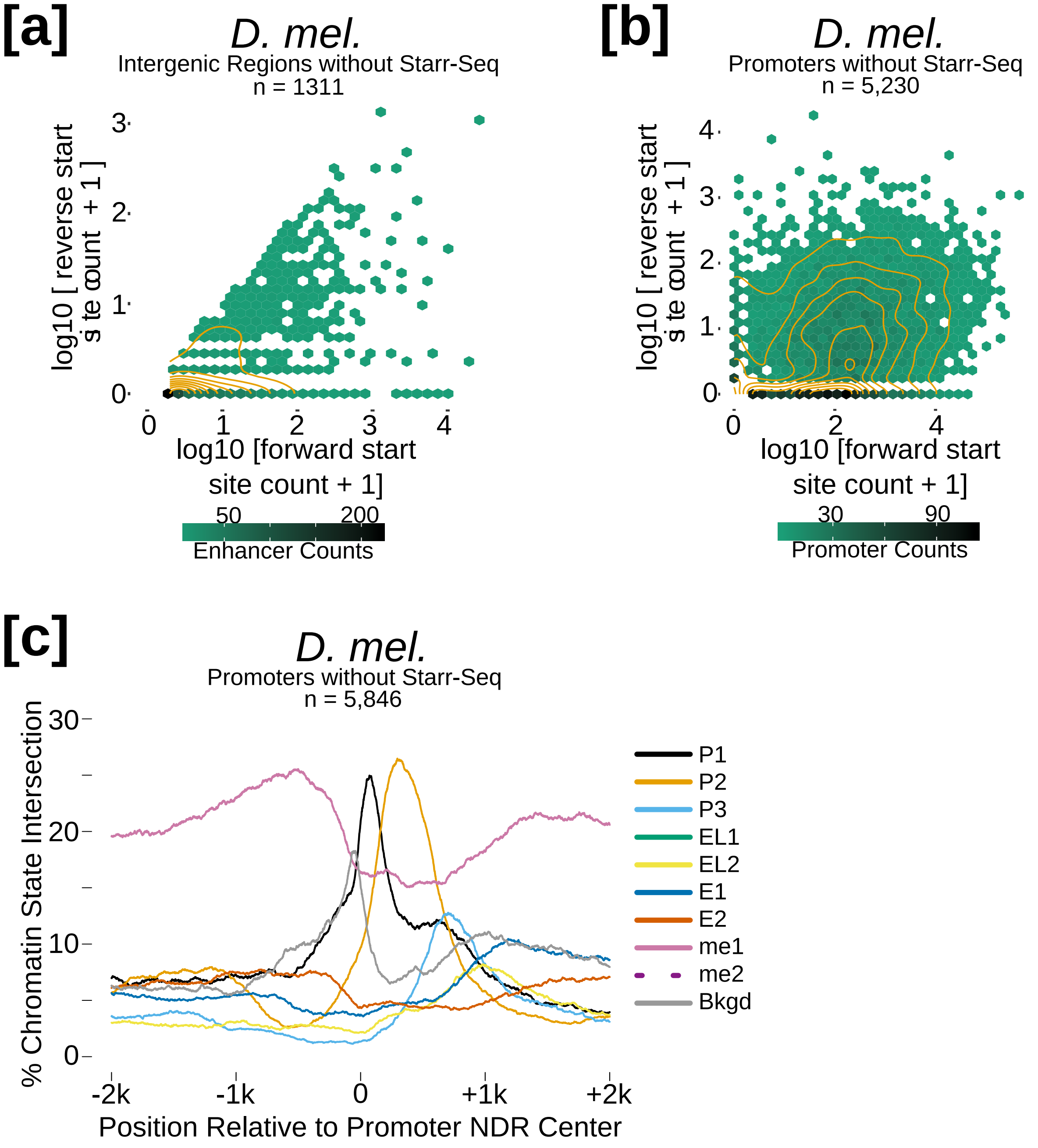

[a]
D. mel.
Intergenic Regions without Starr-Seq
n = 1311
log10 [reverse start site count + 1]
log10 [forward start site count + 1]
50
200
Enhancer Counts
[b]
D. mel.
Promoters without Starr-Seq
n = 5,230
log10 [reverse start site count + 1]
log10 [forward start site count + 1]
30
90
Promoter Counts
[c]
D. mel.
Promoters without Starr-Seq
n = 5,846
% Chromatin State Intersection
Position Relative to Promoter NDR Center
-2k
-1k
0
+1k
+2k
P1
P2
P3
EL1
EL2
E1
E2
me1
me2
Bkgd

Supplementary Figure 5. Transcription initiation directionality in negatively-selected STARR-Seq regions.

a. Forward versus reverse direction PRO-cap counts for intergenic NDRs that *do not* intersect STARR-seq peaks and have at least one count on one strand. **b.** Forward versus reverse direction cap-selected PRO-seq counts for promoter NDRs that *do not* intersect STARR-seq enhancer peaks and have at least one count on one strand. **c.** Chromatin state coverage plot for promoters that do not intersect STARR-seq peaks.

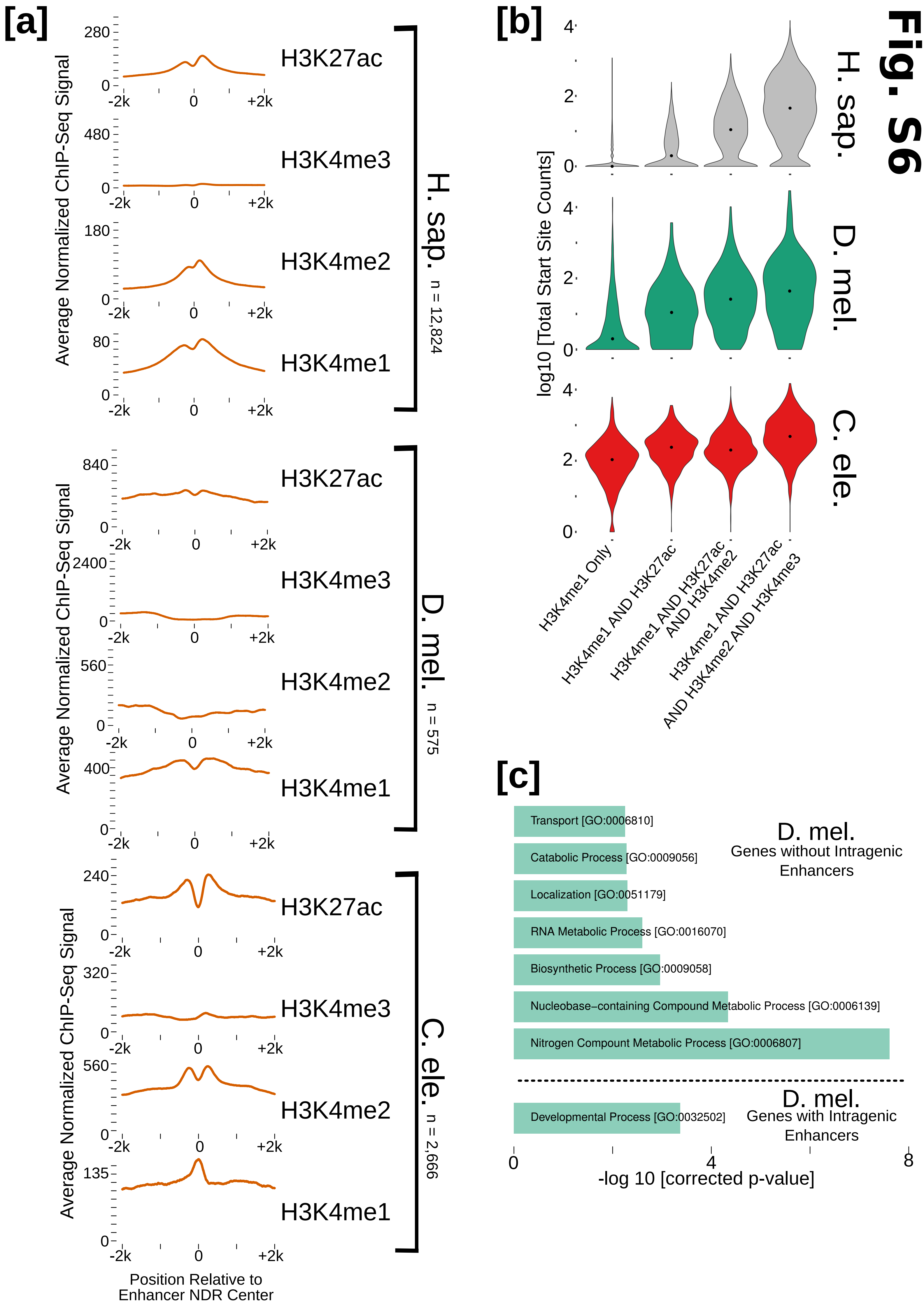
[a]
Average Normalized ChIP-Seq Signal
280
0
-2k
0
+2k
H3K27ac
480
H3K4me3
180
H3K4me2
80
H3K4me1
H. sap.
n = 12,824
840
2400
560
400
D. mel.
n = 575
240
320
560
135
C. ele.
n = 2,666
Position Relative to
Enhancer NDR Center
[b]
log10 [Total Start Site Counts]
4
2
0
H. sap.
D. mel.
C. ele.
H3K4me1 Only
H3K4me1 AND H3K27ac
H3K4me1 AND H3K27ac AND H3K4me2
H3K4me1 AND H3K27ac AND H3K4me2 AND H3K4me3
[c]
Transport [GO:0006810]
Catabolic Process [GO:0009056]
Localization [GO:0051179]
RNA Metabolic Process [GO:0016070]
Biosynthetic Process [GO:0009058]
Nucleobase−containing Compound Metabolic Process [GO:0006139]
Nitrogen Compound Metabolic Process [GO:0006807]
D. mel.
Genes without Intragenic
Enhancers
Developmental Process [GO:0032502]
D. mel.
Genes with Intragenic
Enhancers
0
4
8
-log 10 [corrected p-value]

Fig. S6

Supplementary Figure 6. Distal enhancer NDR characteristics.

a. Average analyses of histone PTMs ChIP-Seq signal relative to midpoints of intergenic NDRs intersecting at least H3K27ac and H3K4me1 peaks. Y-axis heights are set to be the same as for promoter regions (Figure S3C). **b.** Total PRO/GRO-cap count distributions for distal NDRs intersecting H3K4me1 only (n = 12,408(Hs); 802(Dm); 484(Ce)), H3K4me1 and H3K27ac only (n = 1,002(Hs); 284(Dm); 722(Ce)), H3K4me1, H3K4me2 and H3K27ac only (n = 7,324(Hs); 225(Dm); 741(Ce)), or H3K4me1, H3K4me2, H3K4me3 and H3K27ac (n = 7,231(Hs); 119(Dm); 941(Ce)),. Black dots represent median values. **c.** Gene Ontology Biological Process analysis for transcripts that harbor active intragenic enhancers (top) and those that do not (bottom).

Fig. S7

[a]

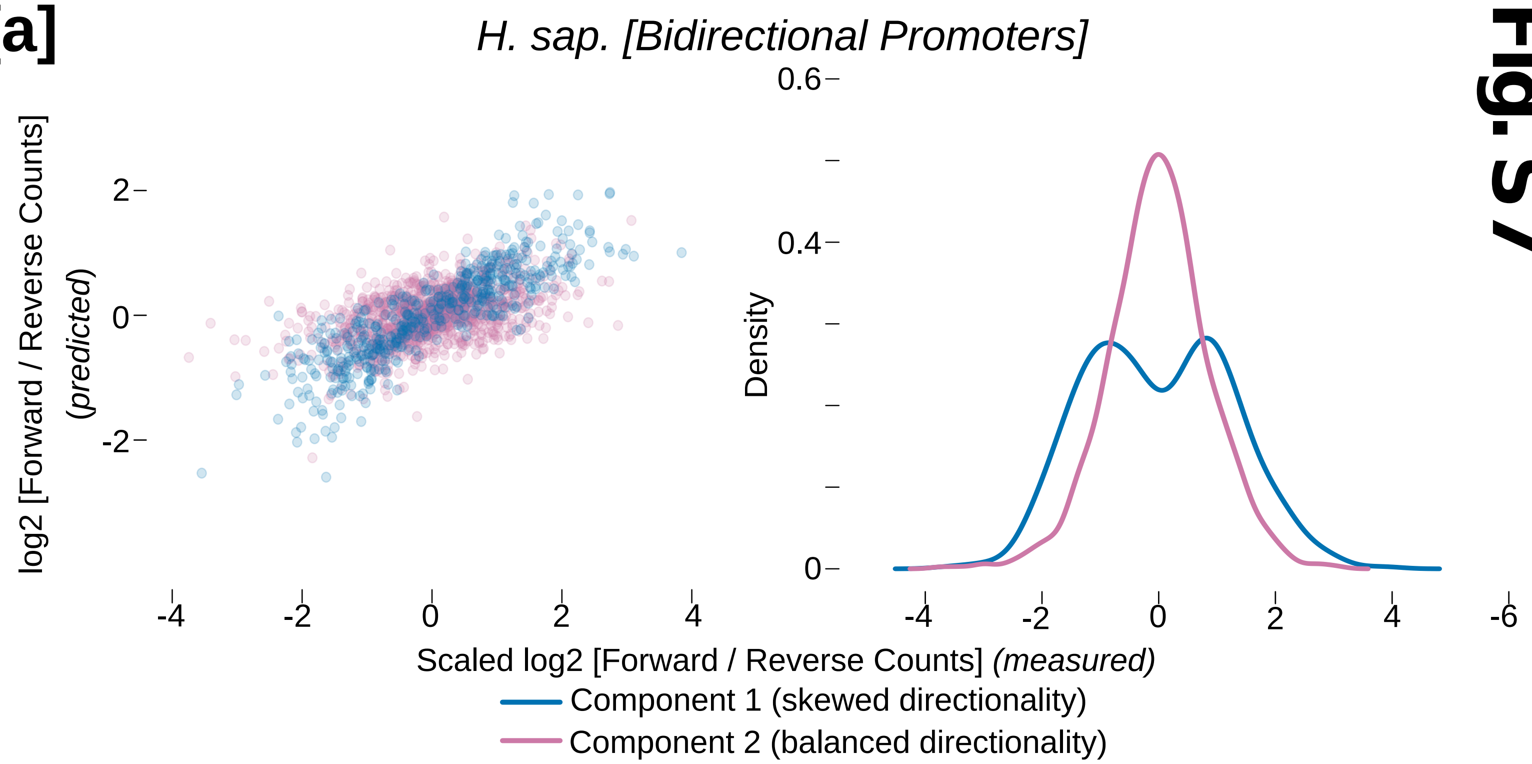

[b]

H. sap. [Divergent Promoters]

	Component 1 (Skewed Directionality)	Component 2 (Balanced Directionality)
Intercept	-0.0169	0.0108
Sequence Score Ratio	0.6686	0.1436
H3K4me3 Ratio	0.1387	0.3758

[c]

H. sap. [Enhancers]

	Component 1 (Skewed Directionality)	Component 2 (Balanced Directionality)
Intercept	0.0189	0.0023
Sequence Score Ratio	0.1831	0.3613
H3K4me3 Ratio	0.7457	0.0413

Supplementary Figure 7. Predictive models of transcription initiation directionality.

a. Same as Figures 6A and 6B but for bidirectional (stable-stable) promoters containing significant forward and reverse levels (n = 1597). Predictions of directionality type and level were made based on the model learned on divergent (stable-unstable) promoters. **b.** Regression coefficients learned when training the model on all promoter regions (same model as in Figure 6A). **c.** Regression coefficients learned when training the model on all enhancer regions (same model as in Figure 6B).

H. sap.						
	ATAC-Seq	Dist. (F)	Dist. (R)	Seq. (F)	Seq. (R)	Express. (F)
Dist. (F)	0.15					
Dist. (R)	0.2	0.26				
Seq. (F)	-0.14	0.16	0.01			
Seq. (R)	-0.10	0.01	0.20	0.12		
Expr. (F)	0.09	-0.26	-0.01	0.45	-0.02	
Expr. (R)	0.09	0.03	0.00	-0.03	0.45	0.23
**D. mel.**						
	ATAC-Seq	Dist. (F)	Dist. (R)	Seq. (F)	Seq. (R)	Express. (F)
Dist. (F)	0.01					
Dist. (R)	0.14	0.14				
Seq. (F)	-0.00	-0.11	-0.02			
Seq. (R)	0.03	0.05	0.14	0.07		
Expr. (F)	0.28	-0.35	-0.07	0.45	-0.02	
Expr. (R)	-0.00	-0.04	0.3	-0.08	0.39	0.09
**C. ele.**						
	ATAC-Seq	Dist. (F)	Dist. (R)	Seq. (F)	Seq. (R)	Express. (F)
Dist. (F)	0.19					
Dist. (R)	0.22	0.21				
Seq. (F)	-0.07	0.24	-0.05			
Seq. (R)	-0.08	-0.02	0.20	0.04		
Expr. (F)	0.29	-0.16	0.02	0.52	-0.03	
Expr. (R)	0.18	-0.04	0.16	0.02	0.50	0.09

Supplementary Table 1.

Full partial correlation table between total PRO/GRO-cap counts (Expr.), ATAC-seq signal, TSS distribution entropy (Dist.) and core promoter sequence score sums (Seq.) in forward and reverse directions for promoter NDRs with significant forward and reverse TSSs.

H. sap.								
	ATAC-Seq	H3K27ac (F)	H3k27ac (R)	H3K4me3 (F)	H3K4me3 (R)	H3K4me2 (F)	H3K4me2 (R)	H3K4me1 (F)
H3K27ac (F)	-0.02							
H3K27ac (R)	-0.03	0.49						
H3K4me3 (F)	0.05	0.41	-0.18					
H3K4me3 (R)	0.03	-0.2	0.47	0.25				
H3K4me2 (F)	0.00	0.12	-0.06	0.18	0.03			
H3K4me2 (R)	0.09	-0.04	0.08	0.02	0.42	0.1		
H3K4me1 (F)	-0.11	-0.21	0.03	-0.21	-0.02	0.61	-0.05	
H3K4me1 (R)	-0.06	0.05	0.17	-0.02	-0.41	0.03	0.5	0.11

D. mel.								
	ATAC-Seq	H3K27ac (F)	H3k27ac (R)	H3K4me3 (F)	H3K4me3 (R)	H3K4me2 (F)	H3K4me2 (R)	H3K4me1 (F)
H3K27ac (F)	0.04							
H3K27ac (R)	0.11	0.34						
H3K4me3 (F)	-0.02	0.49	-0.19					
H3K4me3 (R)	-0.08	-0.1	0.55	0.36				
H3K4me2 (F)	0.17	-0.07	0.04	0.48	-0.06			
H3K4me2 (R)	-0.09	0.02	0.09	-0.18	0.38	0.35		
H3K4me1 (F)	-0.08	-0.01	0.04	-0.37	0.12	0.43	-0.2	
H3K4me1 (R)	0.25	-0.01	0.17	0.22	-0.52	-0.21	0.33	0.49

C. ele.								
	ATAC-Seq	H3K27ac (F)	H3k27ac (R)	H3K4me3 (F)	H3K4me3 (R)	H3K4me2 (F)	H3K4me2 (R)	H3K4me1 (F)
H3K27ac (F)	0.18							
H3K27ac (R)	0.16	0.59						
H3K4me3 (F)	0.12	0.28	-0.15					
H3K4me3 (R)	-0.17	-0.11	0.38	0.25				
H3K4me2 (F)	0.12	0.23	-0.20	0.16	-0.1			
H3K4me2 (R)	-0.00	-0.20	0.26	-0.01	0.19	0.71		
H3K4me1 (F)	0.00	0.08	0.03	0.02	0.15	0.25	-0.05	
H3K4me1 (R)	0.06	0.02	0.03	-0.03	-0.01	-0.03	0.25	0.33

Supplementary Table 2.

Full partial correlation table for active promoter histone PTMs and ATAC-Seq in forward and reverse directions for promoter NDRs with significant forward and reverse TSSs.
